# Service provider perspectives on how COVID-19 and pandemic restrictions have affected intimate partner and sexual violence survivors in Canada: a qualitative study

**DOI:** 10.1186/s12905-022-01683-4

**Published:** 2022-04-11

**Authors:** Sonia Michaelsen, Hervé Djiofack, Elisabeth Nombro, Olivier Ferlatte, Bilkis Vissandjée, Christina Zarowsky

**Affiliations:** 1 École de Santé Publique, Université de Montréal, 7101 Park Ave, Montreal, QC H3N 1X9 Canada; 2 Centre de Recherche en Santé Publique, Université de Montréal et CIUSSS du Centre-Sud-de-l’Île-de-Montréal, 7101 Park Ave, Montreal, QC H3N 1X9 Canada; 3 Faculty of Education, University of New Brunswick, 10 MacKay Drive, Marshall d’Avray Hall, Room 227, P.O. Box 4400, Fredericton, NB E3B 5A3 Canada

**Keywords:** COVID-19, Frontline workers, Intimate partner violence, Sexual violence, Canada

## Abstract

**Background:**

The COVID-19 pandemic may increase risk of intimate partner and sexual violence and make relevant services less accessible. This study explored the perspectives of intimate partner and sexual violence workers across Canada on how the COVID-19 pandemic has affected the survivors with whom they work.

**Methods:**

Using a qualitative descriptive design, we interviewed 17 management and frontline staff of organizations supporting survivors of intimate partner and sexual violence across Canada. Results: We identified 4 themes that describe the impacts of COVID-19 on intimate partner and sexual violence survivors, from the perspective of service providers: (1) No escape; (2) Isolation; (3) Tough decisions; and (4) Heightened vulnerability. These narrative findings are presented first, followed by an analysis within a social determinants of health framework. Interpreting our findings against such a framework revealed a complex interplay of social determinants, notably social support, access to services, and poverty, that produced several challenges for intimate partner and sexual violence survivors during COVID-19.

**Conclusion:**

According to service providers, intimate partner and sexual violence survivors in Canada faced several challenges during the pandemic, including reduced ability to escape their situations, increased isolation, increasingly complex decisions, and heightened vulnerability. Our findings demonstrate the critical need to adopt a broader, more holistic approach in tackling  intimate partner and sexual violence by also addressing socioeconomic issues such as poverty and marginalization.

**Supplementary Information:**

The online version contains supplementary material available at 10.1186/s12905-022-01683-4.

## Background

### Intimate partner and sexual violence

Intimate partner violence (IPV) and sexual violence (SV) are two intertwined public health issues affecting approximately one-third of women worldwide [1]. Since the onset of the current COVID-19 pandemic, activists and experts have warned that confinement measures may lead to a rise in IPV and SV and make it more difficult for survivors to access support and services [2–4]. Preliminary data regarding call volumes to police and helplines from different countries are mixed. Some services have reported an increase in calls while others have reported a decrease [5–7]. One study in Dallas found that calls to a local police station for IPV increased immediately following lockdown measures but then decreased afterwards [8]. These findings suggest that there has been a change in help-seeking behaviours amongst survivors but need careful consideration when interpreted, as a decrease in calls does not necessarily mean a corresponding decrease in violence. Such findings are limited as they offer little insight into the pathways affecting help-seeking behaviours.

Lockdowns and other confinement measures may increase women’s vulnerability to violence and abuse by cutting women off from their social networks. With social gatherings banned and businesses such as restaurants and cinemas closed, there is less opportunity to meet with friends and family. Work from home measures or job losses associated with the pandemic may result in abusers being home much more often, leading to increased opportunities to control their partner [6].

The pandemic has also led to increased unemployment and poverty in many countries [9, 10]. Financial precarity may not only increase stress and conflict within a relationship but also make it more difficult for women to leave their abusive partner [4]. Furthermore, males who subscribe to traditional gender roles, such as believing that men should be the “breadwinners”, may feel emasculated by losing their job [11, 12]. Feelings of inadequacy can increase their need to exert control over their partners in other ways, such as through violence and manipulation [13]. Women in situations of financial precarity are also at greater risk of sexual exploitation which, in turn, increases their risk of sexual violence [14]. During the initial stages of the pandemic, media within the United States reported that some landlords pressured cash-strapped tenants to offer “sex-for-rent” [15]. Finally, to cope with the challenges of the pandemic, individuals may turn to substance use, a well-documented risk factor for violence [16, 17].

However, contacting IPV and SV survivors during COVID-19 and listening to and documenting their experiences may be unethical and dangerous if survivors are unable to share their stories safely and confidentially when constantly at home with their abusers. Researchers have found creative solutions, instead collecting data from public or secondhand sources, including those close to survivors. A qualitative analysis of Reddit (a public online network of communities) forums where survivors posted about their experiences of IPV during COVID-19 revealed that they had a harder time accessing services, that the pandemic interrupted their plans to leave, and that their abusers were using COVID-19 as a control tactic [18]. Informal supporters (e.g. friends, relatives) of IPV survivors in the UK described how COVID-19 and pandemic control measures challenged their ability to provide support to their affected loved ones and that the abuse seemed to intensify during the pandemic [19]. Healthcare workers, lawyers, and social workers in the United States reported similar challenges; survivors had a harder time accessing services, the pandemic exacerbated existing traumas and, as a result, survivors experienced a deterioration in mental health [20].

### A social determinants of health framework

Because of the varying degrees of containment measures implemented by different countries, contextualized research is required to better understand the ways in which COVID-19 and social distancing measures have affected IPV and SV survivors. Developing policies and interventions tailored to specific contexts requires both context-specific and comparative evidence. A social determinants of health (SDOH) framework is useful as it maps readily against existing programming and government structures (such as housing, social services, education, health services) within a given context, which may facilitate interventions at all levels. Conversely, beginning and ending with women’s lived experiences challenges sectoral or institutional boundaries and “social determinant siloes”.

**Fig. 1:**
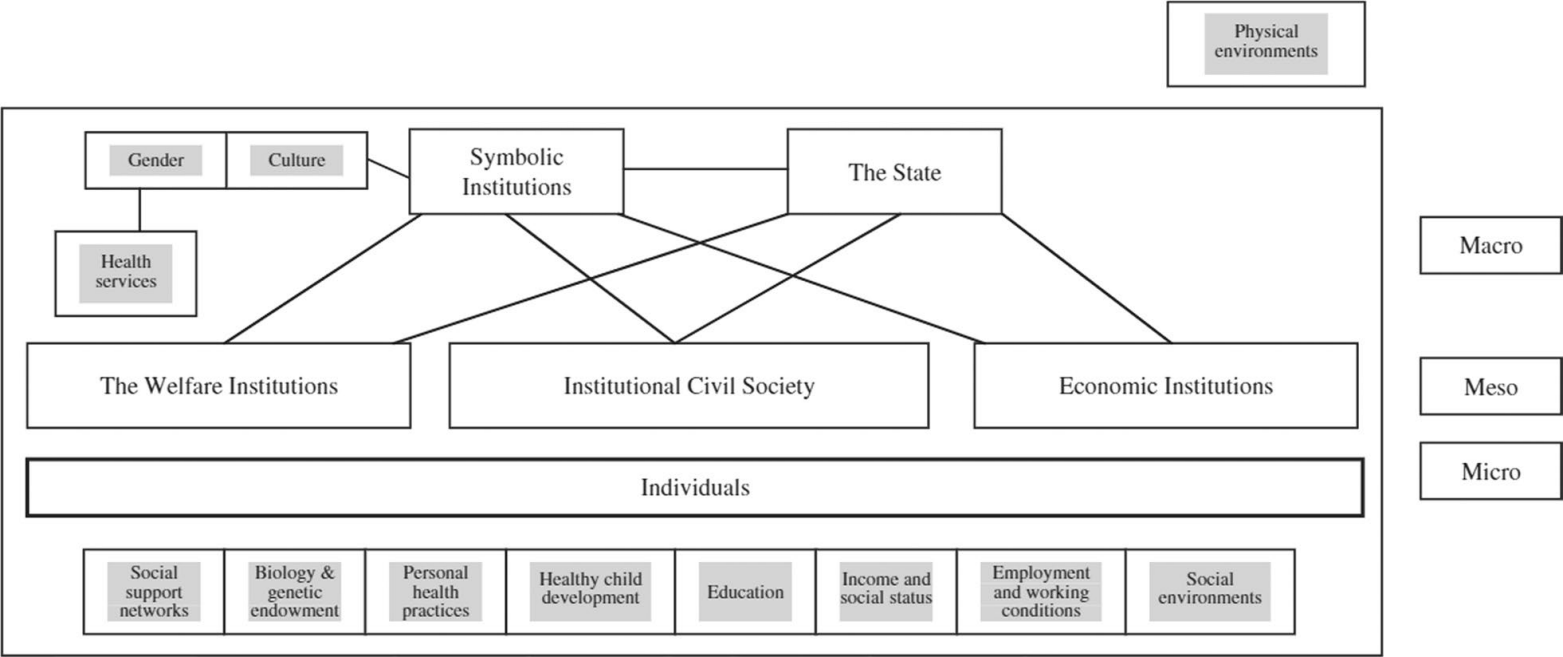
**A** social determinants of health framework, from Thurston and Vissandjée (2005)

Canada has identified 11 broad SDOH, based on the findings of the World Health Organization’s SDOH commission [21]: income and social status, employment and working conditions, education and literacy, childhood experiences, physical environments (geography, built environments), social supports, healthy behaviours, access to health services, gender, culture, and race/racism [22, 23]. These SDOH operate and interact at different levels of the ecosystem within Canada to produce and reinforce inequities (see Fig. 1). At the macro-level are broader laws, policies, and sociocultural norms and ideologies that shape the social, political, and economic institutions—such as health services and community organizations—operating at the meso-level [22, 23]. The micro-level of the SDOH framework refers to the more immediate lived experiences and relationships of an individual, including their social support networks, education, income and employment status, and personal behaviours [22, 23]. In the context of COVID-19, containment measures enacted by state actors at the macro-level may interact with broader social and gender norms to affect not only IPV and SV resources (meso-level) but also women themselves (micro-level).

## Methods

### Study overview

This article explores the perspectives of IPV and SV service providers in Canada, such as shelter workers and counselors, on how the COVID-19 pandemic and associated measures have affected survivors with whom they work. IPV and SV service providers have a unique perspective to offer, as they work with survivors more closely, and over a longer period of time, than many other professionals such as emergency health workers. Building a trusting, ongoing relationship with a number of clients is central to their work [24]. Their perspectives may offer nuanced insights into this complex problem—and on possible context-sensitive strategies to respond to IPV and SV both during and after the crisis phase of this pandemic.

At the onset of the pandemic in Canada, in April 2020, we formed a multidisciplinary team of academic researchers and students with experience in violence against women, trauma and mental health, and public health systems and policies, and practitioners from organizations working in IPV and SV. SM (female), the first author and study coordinator, is a frontline worker at a women’s shelter for IPV and a Ph.D. candidate in Public Health whose research focuses on gender-based violence. SM first conducted key informant interviews with the practitioner members of our team, to explore the issues currently affecting IPV and SV organizations and how to best conduct research with such organizations during a time of crisis. These key informant interviews informed our study design; a qualitative descriptive design utilizing in-depth and semi-structured interviews [25]. Qualitative descriptive research has the goal of providing “a comprehensive summarization, in everyday terms, of specific events experienced by individuals or groups of individuals” [26]. Qualitative descriptive studies adopt a less interpretive approach and stick closer to the actual words and stories within the data and, as such, a strength of qualitative descriptive research is the increased likelihood that they accurately depict the accounts and experiences of participants [27]. Findings from qualitative descriptive studies can therefore be easily understood and applied by both practitioners and policy makers [27]. This study is anchored in pragmatism as an overall framework of inquiry. Pragmatism focuses on lessons that can be drawn from people's experiences and on solutions that can be developed in response to real problems [28]. The key informant interviews also helped inform the development of the interview guide (provided as Additional file 1), which was first piloted with members of our team.

### Participants

Eligible participants included management and frontline staff of IPV and SV organizations within Canada, or from other, related organizations that offered support or programming for IPV and/or SV. Participants had to speak either English or French. A total of 17 individuals participated from 15 organizations including women’s shelters, crisis lines, counselling services, advocacy organizations, victim services, and a youth centre. All participants were women, reflecting the largely female composition of staff working within IPV and SV services. Participant and organizational characteristics are displayed in Table 1. As it is the case for many IPV and SV support organizations, some providers covered the role of both management staff and frontline workers.

**Table 1:** Characteristics of participants and their organizations

Participant	Organization location	Services offered	Job position	
			Managerial	Frontline
Arielle	Central Canada	Crisis line Shelter Psychosocial support	X	
Mia	Central Canada	Advocacy and education	X	
Amelia	Central Canada	Crisis line Psychosocial support	X	X
Marie-Ève	Central Canada	Crisis line	X	
Eloise	Eastern Canada	Crisis line Psychosocial support	X	
Holly	Central Canada	Shelter Psychosocial support	X	
Katherine	Eastern Canada	Psychosocial support		X
Genevieve	Western Canada	Psychosocial support	X	X
Celeste	Western Canada	Psychosocial support	X	X
Jocelyne	Central Canada	Advocacy and education	X	
Pascale	Eastern Canada	Shelter Psychosocial support	X	
Henrietta	Central Canada	Crisis line Shelter Psychosocial support	X	
Solange	Central Canada	Advocacy and education	X	
Letitia	Eastern Canada	Shelter Psychosocial support		X
Suzanne	Eastern Canada	Shelter Psychosocial support	X	
Margot	Eastern Canada	Crisis line Shelter Psychosocial support		X
Dominique	Central Canada	Crisis line Shelter Psychosocial support		X

### Recruitment and procedures

Participants were recruited via purposive and snowball sampling. As the research team was experienced in the field, we first reached out by e-mail to our contacts and colleagues within the field using a brief but detailed script, explaining the purpose of the study and inviting them to participate. Following their participation, we then asked participants if they could put the research team in touch with other potential participants via e-mail, using a script we provided. Through this process, 28 organizations and services were contacted. A total of 17 individuals replied, agreeing to participate. We then followed up with a consent form by e-mail, available in either English or French, and scheduled an interview time. No participant dropped out during the study. Due to social distancing measures and the cross-country nature of the study, we conducted all interviews via Zoom, between June and September 2020. Participants had the option of being interviewed in English or French. We endeavoured to include a diversity of services providing support for IPV and SV (e.g. crisis lines, women’s shelters, indigenous organizations) as well as to have geographical variation (e.g. rural and urban organizations, organizations from different provinces). SM, HD (male, Ph.D. Candidate, Public Health), and EN (female, M.Ed. candidate, Counselling Psychology) began all interviews by introducing themselves, their academic and professional background, and reviewing the purpose of the study. During the interviews, they jotted down notes and impressions. On average, interviews lasted 70 min. They were audio recorded and then transcribed verbatim by HD and EN. Interview questions were open-ended and focused on whether service providers noticed a change in needs amongst their clients, whether service providers noticed a change in the nature of violence amongst their clients, and what challenges, if any, their clients have been struggling with.

### Analyses

Interview transcripts were imported and analyzed inductively in NVivo, using thematic analysis [29]. As a first step, SM read and reread the transcripts to become familiar with the data, noting impressions and initial ideas for codes. Then SM began assigning codes to sentences and extracts, distilling and condensing their meaning. SM formed a provisional codebook relevant to our study objective. As more transcripts were coded and after several read throughs, the codebook was refined and finalized. Initial codes included: alternative locations, reduced availability, travel bans, new control tactics, quarantine, immigrant women, elderly women, poverty, and childcare. Codes with similar meanings were then grouped together into themes. SM sent a sample of 4 transcripts to the research team, for co-authors to read and to discuss initial interpretations of the data and confirm codes and themes. Once the themes were refined, SM reached out to the participants and shared a summary of the findings, along with supporting quotes, in order to receive their feedback, ensure all quotes were accurately interpreted, and to validate the results. A total of 9 participants replied and validated the findings; 3 never replied; and 5 were no longer working at the organization and no alternate contact information was available. Findings were then interpreted deductively against a SDOH framework.

### Ethical considerations

We made a conscious decision to speak with service providers instead of survivors themselves, as the pandemic may be an especially dangerous time for IPV survivors if they are constantly at home with their abusers and may be an especially triggering, traumatic time for survivors to recount their experiences. Recognizing that service providers may be overwhelmed with increased demand and having to adapt their services in the wake of COVID-19, we also waited three months into the pandemic before reaching out to organizations, relying on the advice of the service providers on our team as to when the situation within organizations stabilized. Because interviews were conducted virtually, all participants were given a passcode that was required to access the Zoom call, in order to ensure their privacy and confidentiality. All participants have also been given pseudonyms, and any reference to their organization’s name or geographical location has been removed. All quotes originally in French have been translated into English. We have not indicated which quotes have been translated to further protect anonymity, as not all provinces in Canada commonly speak French. In addition, because some Canadian provinces may only have one provincial-wide service such as SV crisis line, service providers are referred to by their broader geographic location (i.e. eastern, central or western Canada). This study was approved by the Comité d’éthique de la recherche en sciences et en santé (CERSES) at the Université de Montréal (20-079-D-2020-942).

## Results

We identified 4 themes that describe the perspectives of service providers on the effects of the COVID-19 pandemic on IPV and SV survivors: (1) No escape; (2) Increased isolation; (3) Facing complex decisions; and (4) Heightened vulnerability. These findings are presented first, followed by an analysis against a social determinants of health framework.

### Providers’ perspectives on survivors’ experience

#### No escape

Many participants stressed the ways in which pandemic-related measures, including stay-at-home orders, the closure of restaurants and other businesses, and travel bans, made it increasingly difficult for survivors to flee violence. COVID-19 introduced obstacles at different points along the help-seeking trajectory, from the initial stage of reaching out, to making a safety plan, to actually leaving.

Because a defining feature of abusive relationships is control and isolation, opportunities for survivors to reach out for help were already limited in pre-pandemic times. Henrietta, a management staff, describes how these opportunities were further reduced when service industries closed their doors and work-from-home measures were implemented:

Women call us when their partners are at work or they call us when they're on their lunch break at work. They call us when they're at the hairdresser, that's actually a big one. Sometimes the degree of coercive control that partners have over their spouses, their partners, the women, hairdressers are like one of the only things they are allowed to do on their own. And so when all of that stopped and there's no hairdresser to go to, women just didn't have the opportunities to call us. They're stuck at home.

Even if a woman was able to find a moment where she could safely and privately contact an organization such as a shelter for help, it was not guaranteed they would be able to accept her. Several participants from shelters explained that, even before COVID-19, demand for shelter space exceeded availability. Participants reported that limits in availability were exacerbated during the pandemic as some shelters had to reduce or completely stop new intakes in order to ensure adequate space for social distancing, to protect an existing resident who was immuno-compromised, or because they had insufficient staff to take on additional clients. Marie-Eve, a management staff, explained that the situation was particularly dire at the onset of the pandemic:

Sometimes, unfortunately, really unfortunately, we have to tell women there is no space. And that’s what happened at the start of COVID. At the start of COVID, shelters didn’t know how they were going to deal with this. They already had women and children there who they had to protect, and they couldn’t easily accept someone if they didn’t know [their COVID status]. So there was a lot of shelters that said before we know how to organize ourselves, we’re not accepting anyone. And then we found ourselves in a situation where, within a few days, there was almost no availability across [the province].

Without any safe place to go, this not only meant that survivors of IPV may have remained trapped in an abusive relationship or that survivors of SV felt unsafe at home, but that those who reached out and were turned away may have become discouraged from reaching out for help again in the future.

According to service providers, the pandemic also created additional hurdles when it came to safety planning. Shelter workers described that they typically make a careful plan with survivors to leave in a way that does not alert their abuser to their intentions. However, they noted that with many places closed due to the pandemic, survivors had difficulties finding reasons to leave the house without raising suspicion:

So if someone's calling and they live with her abuser, a lot of the times they could go to, let's say, Tim Hortons [a local coffee chain] or something and then we could send a cab there to get them, okay? Where in the beginning of this, there was nowhere really for them to go that wouldn’t incorporate like a red flag [to their abuser] (Holly, management staff).

Other survivors, instead of choosing to go to shelter, prefer or have the option to stay with friends or family. However, even if a woman was able to pack a bag and leave, her escape could be impeded by the inter-region travel bans imposed by many provinces in Canada:

So we’ve seen something interesting where, you know, one person was fleeing violence in [one province] and wanted to go to [another province] to be with their mom or be with their parents but they faced a challenge actually crossing at the [provincial] border and they were turned back. It’s this interesting dynamic we’re also starting to see around interprovincial, you know travel across Canada, across borders. It’s been difficult and we’ve seen it with some of the survivors trying to cross to get to a safer location they’ve been stopped.

Leaving a violent situation can be both emotionally and logistically difficult, and the pandemic increased these complexities. Work-from-home measures and the closure of many businesses such as restaurants and salons meant survivors had less opportunity to reach out to services or to seek shelter in a safe, carefully planned way. Travel bans exacerbated this issue. Confinement measures therefore may have inadvertently kept many women in dangerous situations for longer and potentially discouraged some from attempting to leave in the future due to the discouraging, difficult process during the pandemic.

#### Increased isolation

The increased challenges in fleeing violence were compounded by increased isolation. Most participants described how survivors were struggling with isolation during the pandemic, caused both directly by social distancing and confinement measures, and indirectly through increased controlling behaviours by partners and reduced number of clients in shelters. Confinement measures such as work-from-home directives and bans on social gatherings meant survivors had far fewer opportunities to leave their house and spend time with their usual sources of social support:

And then obviously just that isolation piece of it. Like that one's just huge for us, what we're seeing is, even if it's not pursuing shelter, right, when you're in an abusive situation, you can at least go to work, spend time with your friends or your family or, you know, get out, even go to Tim Hortons [a local coffee shop] or whatever (Holly, management staff).

At the same time, COVID-19 presented an opportunity for abusers to exert their control and further limit survivors’ contact with others. Under the guise of health and safety, abusive partners could argue that it was not safe to leave the house, to go to public places, or to interact with other people. Dominique, a frontline worker, explained:

[…] If you have a controlling partner and then, with COVID, you become a little bubble within your family and there are all these micro-decisions to agree upon now—“Are we going to do the groceries every 2 weeks, or every 3 weeks? Are we going to go to restaurants or not? Are we going to see friends or not?” So, all these micro-decisions which are already often a form of control when there is abuse. So, now there’s even more justification for control.

This meant that even activities that were permissible under public health guidelines, such as going to the store or taking a walk outdoors with a friend, could be difficult for survivors to undertake. Participants expressed concern about the effects of this heightened isolation on survivors, noting that they were a group who were already typically isolated in pre-pandemic times. Feeling alone could worsen the anxiety, depression, and post-traumatic stress disorder that many survivors struggle with and, ultimately, make it even harder for them to leave an abusive relationship by feeling like there are no other options available.

Even if women managed to leave their abusers and safely make it to shelter, they often still found themselves isolated from the usual forms of support they would typically receive there in pre-pandemic times. Participants described breaking isolation as a key strategy when working with survivors, but COVID restrictions limited their ability to do so. Some shelter workers noted that, in order to reduce the risk of transmission, they implemented a rule where every newly arrived woman had to quarantine for 14 days. Henrietta, a management staff, explained how this meant there were far fewer opportunities for women to connect with workers and build a trusting, open relationship that is usually a central feature of their initial days in shelter: “being in quarantine those first two weeks are really hard. So, like building that alliance and connection is more challenging than it is when you can, you know, pop into the office”. Henrietta further recounted that women often benefit not only from formal support within a shelter environment but from informal support as well, by developing peer relationships with other women. However, due to social distancing measures, communal spaces such as living rooms and kitchens were now closed, meaning women were less able to interact with each other:

[…] it's just such a different kind of shelter, like not being able to sit around the dinner or lunch table together. Women are not benefiting from that other layer. We never presume to be, like, the counselling relationship is one benefit, but being in this space, and for the first time, women realising that they're not alone… (Henrietta, management staff).

There was often a tension among participants between protecting the health of survivors and fostering social connection amongst them. Some shelters moved to a hotel during the pandemic, in order to be able to ensure adequate physical distancing. Holly, another management staff, describes how many survivors struggled with this move as it meant that they were now spending most of their time alone in a hotel room, contributing to feelings of aloneness:

Some of them have already been isolated for several months or even years with a partner, and then they enjoy that peer support that they get from one another in shelter. And then when we relocated [to a hotel], it kind of took that peer support and social support away in their eyes.

The negative consequences of the interruption of communal life were exacerbated by the fact that several shelters also reduced their intake capacity during the pandemic. Not only were there fewer opportunities for socialization, there was also just fewer women to connect with: “then also the reduction of the beds that are available… that's really a pity because mutual aid can really help women in their journey, after they leave a difficult relationship” (Dominique, frontline worker).

#### Facing complex decisions

These many challenges and obstacles introduced several new, increasingly complex choices for survivors to make that were highlighted by service providers. Participants described how some of these decisions centered around childcare and custody agreements, reaching out for help, and deciding to go to shelter. Many of the guidelines put in place, particularly at the start of the pandemic, lacked nuance and consideration towards the unique situation of survivors. Dominique, a frontline worker, cited custody arrangements as an example:

You know, at the start, the government said that custody arrangements had to be maintained regardless of the situation. There were women who were not comfortable, who had joint custody, but who didn’t feel comfortable sending their children to their husband, their partner, or their ex-husband or ex-partner—the dad—because the dad wasn’t respecting public health guidelines.

Several participants mentioned that abusers were using spreading COVID-19 as a threat, as a way to control survivors, which could make it incredibly challenging for mothers to willingly send their children to their fathers. Mothers faced the difficult decision of either sending their children to the father and potentially putting the children’s, as well as her own, health at risk or face the legal repercussions of disobeying their official custody arrangement.

Protecting one’s health also arose as a central concern in women’s decision-making process on whether to leave an abusive partner or not during the pandemic. For many survivors, going to a shelter is one of the only options available should they choose to leave an abusive relationship, as they may not have the financial means to live on their own or have friends and family nearby to stay with. However, shelters are inherently a communal living space, meaning the risk of transmission there is higher. As Holly succinctly explained, women were faced with the choice: “It's like, do I stay at home and risk my safety, or do I pack up my children and come to shelter and risk our health and safety?”.

Lockdown measures and what these would mean for life within a shelter environment further complicated women’s decision-making process on whether to go shelter or not. Marie-Eve highlighted how, even in pre-pandemic times, leaving is a difficult process where women have to weigh several factors. The pandemic introduced additional considerations due to the heightened uncertainty:

You know, when a woman tries to decide “Do I leave?”, she has a balance. But this balance, it doesn’t have 2 sides, it has about 60 sides. And if you move something, you know, this balance is very complex […] “Will there be a second wave? Will we return to lockdown, and do I want to be in a shelter during lockdown?” […] “If there’s a lockdown, will they send us back home? Will they have to close?

Marie-Eve further explained how this decision to leave was made increasingly difficult for mothers with young children:

I think [COVID-19] will delay the reconsideration of relationships for some victims because it just complicates the situation. You know, will kids be in school next year? In what form? And if I’m in a shelter or an apartment…. If I have a house with a yard, I’m lucky, very lucky, but if I separate [from my partner] and go to a one room apartment, lockdown will be awful, especially if I have two kids, no yard.

While leaving an abusive situation may protect survivors (and their children) from further violence, it can also increase other stressors and this was particularly true during the pandemic. Being in a shelter during lockdown could mean being confined to a single room in an unfamiliar environment, instead of within the familiarity of one’s own home. If there are children involved and schools and daycares closed, this could mean the added burden of childcare in a confined environment.

Finally, one worker described how her team noticed that SV survivors who struggled with trauma but who were not in immediate danger were making the difficult decision to deprioritize their own needs, recognizing that it may be an especially risky time for IPV survivors in particular:

[…] survivors of sexual assault were telling [my team], like, “I'm not a priority right now, you guys go deal with the women who are actually at risk. It's okay.” But what they meant by that is that there was a ton of media locally and provincially that was really focused on domestic violence and intimate partner violence and just how we knew that it was going to increase in scale because of all of the trigger factors that physical distancing involved […] I think there was such a gross internalization of that and like a hierarchicalization [sic] of trauma that happened for some of our survivors (Arielle, management staff).

#### Heightened vulnerability

Almost every participant pointed to how “overall, COVID has made the vulnerable more vulnerable” (Henrietta, management staff). They described how many of the usual risk factors for violence, abuse, and exploitation were being exacerbated by the pandemic, including financial precarity, social exclusion, and language barriers. These same risk factors also made it increasingly difficult for survivors to access IPV and SV services.

Participants mentioned how they noticed many of their clients were struggling financially during the pandemic. Jocelyne, a management staff, explained how these financial challenges could be compounded by women’s immigration status:

[…] a lot of non-status [immigrant] women were not eligible for support through the economic, you know, the emergency response, that's a major one […] because it was dependent on SIN [social insurance] number, because it was dependent on [immigration] status and things like that.

Participants described how financial precarity or poverty not only affected women’s ability to purchase everyday basics such as food, but also intersected with their experiences of social isolation and their ability to reach out for support since many services had moved online during the pandemic:

[…] but again, because [this town] has so much poverty, few people have a cell phone. Very few people have access to Internet. There's no public phone [in this town] or a place where they can go to make calls or to receive calls (Celeste, management staff/frontline worker).

This sudden transition to a virtual world also proved difficult for older people. Katherine, a frontline worker, explained that many older people didn’t necessarily feel comfortable using the technology now required to communicate with social services:

Since COVID I'm like, most of my clients were older, they were going up to their 60s and 70s, so that was new. I think that's because everything was closed and these people didn't know how to access the services anymore, because they didn't know either the phone numbers or the phone numbers were confusing. You called mental health, it told you to leave a message, and like all these things were confusing all of a sudden for the older generation and accessing things online and being able to do meetings through Zoom.

As older people are a group that often face social isolation in pre-pandemic times, this heightened difficulty in accessing needed social support is particularly concerning. Another participant described another group of women who often struggle with isolation and social exclusion: immigrant women with language barriers also faced heightened vulnerability during the pandemic. With many translation services temporarily closed, they were unable to properly communicate with IPV or SV services during a time when they may have needed to the most:

Our immigrant population normally is about 33% of our clients and even now, so like from February to now, it's about 2%, which is not normal […] An immigrant woman who doesn’t speak a lot of English won’t be able to reach out to us without a community service to support them and they’re not there (Genevieve, management staff/frontline worker).

As these different social groups were experiencing increased financial challenges and isolation, participants also explained that many survivors were struggling with more intense violence. Participants described women calling them while sobbing hysterically, locked in their bathrooms, or in the context of police interventions. One worker details the sudden increased severity of physical violence she witnessed amongst clients: “But like the bruises, they went from being fingerprints to actual handprints and broken teeth. So it’s basically slaps turned into punches and bruised ribs” (Letitia, frontline worker). Thus, the increased challenges in reaching out to IPV and SV services amongst certain groups of women coincided at the same time as when these services may have been most needed, as many situations of abuse seemed to be escalating.

### A social determinants of health framework

Our findings reveal a complex interplay of SDOH at the macro, meso, and micro levels that produced several challenges for IPV and SV survivors during COVID-19 (see Table 2 for a summary). It is important to highlight that the COVID-19 pandemic did not suddenly create gender-based violence. Rather, measures put in place by provincial and federal governments and the effects of the pandemic converged with patriarchal social norms and gender dynamics to produce a context that legitimises men resorting to violence and control as a way to cope with the added stress of the pandemic. Other macro-level measures such as the closure of daycares and schools interacted with gender norms around childcare and the burden of care to produce additional challenges for those survivors with children. Survivors with children not only had the added responsibility of taking care of their children during the weekdays but may have prioritized the comfort of their children over their own personal safety by choosing to stay at home instead of going to shelter.

**Table 2:** Findings according to a social determinants of health framework

Level of ecosystem	Social determinant of health	Example
Macro	Gender: gender norms and dynamics Culture: patriarchal society	Escalating abuse: “So a lot of the women I worked with before [COVID] who I was talking to on a regular basis, like maybe once a week or once a month, they all of a sudden, the abuse changed, the abuse got worse because of the stress levels.” (Katherine)
Meso	Access to health and social services	Increased difficulty accessing services that have moved to online/phone due to technological barriers: “[…] but again, because [this town] has so much poverty, few people have a cell phone. Very few people have access to Internet. There's no public phone [in this town] or a place where they can go to make calls or to receive calls” (Celeste)
Micro	Income	Increased financial precarity: “People are struggling financially for food and just basics” (Arielle)
	Social status	Increased vulnerability due to language barriers hindering the ability to access services: “An immigrant woman who doesn’t speak a lot of English won’t be able to reach out to us without a community service to support them and they’re not there” (Genevieve)
	Physical environment: built environment	The built environment of hotels limiting social support: “In all honesty, there's been a little bit of a disconnect working at a hotel. Right. Because they were all in each other's space or in the same house [before]. Like, we all eat lunch at the same table. There's no staff room. We have a staff in an office, obviously, but we're all just in the same space everybody else. And being in a hotel, it's like, well, we're so separated from everyone.” (Holly)
	Social networks	Increased isolation due to lockdown measures: “[…] when you're in an abusive situation, you can at least go to work, spend time with your friends or your family are, you know, get out even go to Tim Hortons or whatever and you couldn't do that” (Holly)

Containment measures at the macro public policy level also affected social determinants at the meso level, particularly access to health and welfare institutions. For instance, public health guidelines centered around physical distancing indirectly affected access to IPV, SV, and other social services as such services transitioned to working from home, providing services online, and/or reducing their intake capacity, thereby making them less available and less accessible. This reduced accessibility was often mediated by social status (micro-level) and geography (meso-level). Individuals living in poverty were not necessarily able to afford the requisite technology and those living in rural areas did not always have access to a proper internet connection in order to participate in this newly virtual world.

Containment measures at the macro-level also affected social determinants at the micro level, including income and social support networks. Individuals were struggling financially, reflecting the mass layoffs that occurred in Canada during COVID-19, as many businesses and services were forced to close their doors. Again, this situation of financial precarity was mediated by social status, as not all survivors were eligible for emergency social assistance due to their immigration status (or lack thereof). Pandemic-related measures also both directly and indirectly affected survivors’ access to social support networks. Bans on social gatherings and inter-region travel prohibitions meant survivors were cut off from friends and families. The closure of businesses such as cafes, restaurants, and beauty salons meant survivors had few opportunities to leave their home and interact with others. At the same time, in order to ensure adequate physical distancing, some shelter services moved to hotels. The built environment, or layout, of hotels meant survivors no longer had access to shared communal spaces where they could connect and form peer relationships with other survivors.

While not an explicit social determinant of health identified by the Canadian government, it is important to highlight the effects of the pandemic, associated measures, and the resulting challenges on survivors’ mental health. While mental health issues can have genetic or neurobiological dimensions, in this particular context we feel it is especially important to distinguish mental health from medical/biological determinants of health. Every single participant described how each of these intersecting factors (the risk of contagion, reduced social support, less accessible services, financial precarity, escalating abuse) worsened the mental health of survivors by increasing stress, fear, and emotional distress and exacerbating pre-existing trauma. In turn, poor mental health can affect many of these social determinants. Increased anxiety and depression can make it harder for a survivor to reach out to their social support networks, access services, and maintain a job. As a result, survivors may become increasingly emotionally and financially dependent on their abusers.

## Discussion

In this study, we interviewed IPV and SV service providers in Canada to better understand how COVID-19 and confinement measures were, from their perspectives, affecting IPV and SV survivors. Our findings corroborate several of the suspected risk factors and pathways proposed by experts at the start of the pandemic [30, 31]. According to participants, their clients struggled with increased financial precarity, childcare burdens, and isolation during COVID-19 while simultaneously facing increased controlling behaviours and new abuse tactics at the hands of their abusers. Participants also detailed how IPV and SV survivors experienced greater mental health needs yet encountered greater challenges in accessing both informal and formal forms of support. Even in non-crisis situations, it is a difficult process for survivors to report their experiences, seek support, and/or leave an abusive relationship. They may feel ashamed, risk being blamed or not believed, be financially dependent on their abuser, or face pressure from their families or religious communities to stay with their partner [32–34]. What our findings suggest is that the pandemic may have worsened survivors’ circumstances and made it *even harder* to seek support than before, both by exacerbating pre-pandemic challenges (e.g. heightening isolation, amplifying vulnerabilities) and by introducing new obstacles (e.g. services moving online or shutting down, travel bans). These findings are concerning as previous research has shown that women who have unsuccessfully attempted to access external support are more likely to stay in an abusive relationship than women who successfully received support [35]. Further complicating the situation is the additional considerations the pandemic and containment measures have imposed on survivors when deciding whether to seek support and/or go to shelter, including whether they or their children will be exposed to COVID-19 there, whether they will have to spend two weeks alone in quarantine upon arrival, and whether a room in an unfamiliar environment would be best for them and their children if schools and daycares remain closed. Our findings also suggest that another difficult consideration that seemed to weigh on some women was whether their situation was important or serious enough to warrant seeking support during the pandemic. Recognizing that services may be overwhelmed during COVID-19, survivors who were not presently experiencing violence but still struggling with trauma may have felt guilty or burdensome reaching out. The accumulation of each of these novel concerns may have prevented many survivors from deciding to access the support they rightly deserve. In the barriers model for IPV developed by Grigsby and Hartman [36], women face several barriers at different levels of the eco-system including psychological barriers, environmental barriers, and societal barriers. The more barriers that exist at different levels of the eco-system, the more complicated it is to extricate oneself from a life of violence.

Most importantly, our findings suggest that social status, including immigration/citizenship status, socioeconomic status, and age, played a crucial mediating role in shaping survivors’ pandemic experiences. Those who were already more likely to experience marginalization in pre-pandemic times were particularly affected by the pandemic and associated measures. Applying a social determinant of health and socio-ecological lens illustrates how the lived, interpersonal experiences of IPV and SV survivors are the result of intersecting social, economic, and political forces. Experiences of IPV and SV also converge with other social and economic issues, including poverty, immigration status, and social exclusion. In order to successfully tackle IPV and SV, addressing these other socioeconomic issues is critical.

Lastly, a novel finding that emerged from this study is the potential impacts of utilizing hotels as a form of shelter for vulnerable populations on their mental health and well-being. Both the media and scholarly articles have highlighted how the pandemic spurred new approaches to housing survivors of domestic violence and homeless individuals in several countries, including the use of hotels, convents, and AirBnBs [37–40]. However, to date, there have been few empirical studies that have explored the experiences of residing in these alternative locations during COVID-19. To our knowledge, the only study that has done so was conducted in Canada by Mantler and colleagues [41]. They found that women appreciated the freedom and independence that hotels afforded them, but that it was more difficult for them to connect with counselors and, for women with children, having access to just one room for multiple people was challenging. Our findings echo these, where service providers described the isolation and loneliness women experienced staying in hotels, as they were no longer able to develop peer relationships with other survivors.

### Recommendations and future directions

This study has several implications for both research and practice. As our findings demonstrate, even if services remain open, survivors may face particular challenges in accessing them. New and creative ideas are therefore needed to improve women’s ability to access support. For example, at the start of the pandemic, France initiated a project to assist IPV survivors by setting up assistance points at supermarkets and pharmacies across the country [42]. Increased partnerships and collaborative efforts between IPV/sexual assault services and other essential services that remain open can not only increase the likelihood of being able to support survivors but can also sensitize other members of the general public to the realities of IPV and SV. It may also be helpful for IPV and SV services to increase efforts to inform the public if their services remain open during the pandemic (e.g. through social media and radio announcements), and to ensure that extra support, such as support groups with women, is available virtually or through the phone to combat the isolation that may come with quarantine measures or staying in alternative locations such as hotels. However, our findings also demonstrate the critical need to adopt a broader, more holistic approach in tackling IPV and SV that goes beyond merely providing shelter and counseling spaces by addressing other social and economic issues including poverty and social exclusion, and broader sociocultural norms surrounding gender and interpersonal dynamics. As our findings suggest, availability of IPV and SV-specific services does not guarantee their uptake, as technology, poverty, language barriers, and imposed isolation all hinder their accessibility. Once it is safer to do so, interviews with survivors could offer additional insights into their experiences during the pandemic, including coping mechanisms, survival strategies, their perspectives on how to best support them in the event of additional waves or a future pandemic, and on how to prevent violence in the first place.

### Limitations

This study has several limitations. We did not manage to recruit participants from every province and territory within Canada. It is therefore possible that service providers within these unrepresented provinces and territories experienced different impacts on their clients that we did not capture. However, in our sample, we did successfully recruit participants from Eastern, Central and Western Canada and attained data saturation. We also reached service providers in both rural and urban areas, as well as service providers for indigenous populations.

Another limitation is the fact that these findings are based on the experiences and perceptions of service providers, rather than from survivors themselves. However, as indicated by our findings, the pandemic may also be a dangerous and traumatic time for survivors and asking them to recount their experiences during the height of a crisis is cruel and potentially damaging. We therefore feel we have struck a balance between being ethical and responsible while still being able to get a sense of how COVID-19 may be affecting survivors.

## Conclusion

According to service providers, IPV and SV survivors in Canada faced several challenges during the pandemic, including reduced ability to escape their situations, increased isolation, increasingly complex decisions, and heightened vulnerability. Our findings demonstrate the critical need to adopt a broader, more holistic approach in tackling IPV and SV by also addressing intersecting macro, meso, and micro-level social determinants of health. We expect that our findings will guide practitioners and policy makers in Canada and elsewhere on how to improve support for IPV and SV survivors, particularly during times of confinement and other social restrictions.

## Supplementary Information

**Additional file 1.** Interview Guide (Interview guide used with the 17 participants)

## Data Availability

The data that support the findings of this study are not openly available due to the risks in identifying participants as true anonymization would be difficult to guarantee, but subsets of the data are available from the corresponding author; SM, upon reasonable request.

## Abbreviations

IPV: Intimate partner violence
SDOH: Social determinants of health
SV: Sexual violence

## References

[1] Violence Against Women [internet]. World Health Organization; 2021. https://www.who.int/news-room/fact-sheets/detail/violence-against-women. Accessed 22 March 2021.

[2] Evans ML, Lindauer M, Farrell ME (2021). A pandemic within a pandemic: intimate partner violence during COVID-19. N Engl J Med.

[3] Harrison E, Giuffrida A, Smith H, Ford, L. Lockdowns around the world bring rise in domestic violence [internet]. The Guardian; 2020. https://www.theguardian.com/society/2020/mar/28/lockdowns-world-rise-domestic-violence. Accessed 23 April 2020.

[4] How COVID-19 could impact victims/survivors of violence [internet]. Minnesota Coalition Against Sexual Assault; 2020. 2 p. https://www.mncasa.org/wp-content/uploads/2020/03/How-COVID-19-Could-Impact-Victims-of-Violence.pdf. Accessed 20 April 2020.

[5] Kourti A, Stavridou A, Panagouli E, Psaltopoulou T, Spiliopoulou C, et al. Domestic violence during the COVID-19 pandemic: a systematic review. Trauma Violence Abuse. 2021. 10.1177/15248380211038690 PMC10011925 34402325

[6] Leslie E, Wilson R (2020). Sheltering in place and domestic violence: evidence from calls for service during COVID-19. J Public Econ.

[7] Perez-Vincent S, Carreras E, Gibbons M, Murphy T, Rossi M. COVID-19 lockdowns and domestic violence: evidence from two studies in argentina [internet]. Inter-American Development Bank; 2021. 48 p. https://publications.iadb.org/publications/english/document/COVID-19-Lockdowns-and-Domestic-Violence-Evidence-from-Two-Studies-in-Argentina.pdf. Accessed 20 Oct 2021.

[8] Piquero AR, Riddell JR, Bishopp SA, Narvey C, Reid JA, Piquero NL (2020). Staying home, staying safe? A short-term analysis of COVID-19 on Dallas domestic violence. Am J Crim Justice.

[9] Kawohl W, Nordt C (2020). COVID-19, unemployment, and suicide. The Lancet.

[10] Valensisi G (2020). COVID-19 and global poverty: are LDCs being left behind?. Eur J Dev Res.

[11] Renzetti CM. Economic stress and domestic violence [internet]. Harrisburg: National Online Resource Center on Violence Against Women; 2009. https://vawnet.org/material/economic-stress-and-domestic-violence. Accessed 22 March 2021.

[12] Forret ML, Sullivan SE, Mainiero LA (2010). Gender role differences in reactions to unemployment: exploring psychological mobility and boundaryless careers. J Organ Behav.

[13] Schneider D, Harknett K, McLanahan S (2016). Intimate partner violence in the Great Recession. Demography.

[14] Peterman A, Potts A, O’Donnell M, Thompson K, Shah N, Oertelt-Prigione S, van Gelder N. Pandemics and violence against women and children [internet]. Center for Global Development; 2020. 45 p. https://www.cgdev.org/sites/default/files/pandemics-and-vawg-april2.pdf. Accessed 2 July 2021.

[15] Woods A. US landlords trying to coerce cash-strapped tenants into ‘sex-for-rent’ agreements [internet]. New York Post; 2020. https://nypost.com/2020/04/17/landlords-sexually-harassing-tenants-during-coronavirus-crisis/. Accessed 25 April 2020.

[16] Abbey A (2002). Alcohol-related sexual assault: a common problem among college students. J Stud Alcohol.

[17] Graham K, Bernards S, Munne M, Wilsnack SC (2008). Unhappy hours: alcohol and partner aggression in the Americas.

[18] Lyons M, Brewer G. Experiences of intimate partner violence during lockdown and the COVID-19 pandemic. J Fam Violence. 2021. 10.1007/s10896-021-00260-x PMC7908951 33654343

[19] Gregory A, Williamson E. ‘I think it just made everything very much more intense’: A qualitative secondary analysis exploring the role of friends and family providing support to survivors of domestic abuse during the COVID-19 pandemic. J Fam Violence. 2021. 10.1007/s10896-021-00292-3 PMC8236317 34219912

[20] Williams EE, Arant KR, Leifer VP, Balcom MC, Levy-Carrick, et al. Provider perspectives on the provision of safe, equitable, trauma-informed care for intimate partner violence survivors during the COVID-19 pandemic: a qualitative study. BMC Women’s Health. 2021; 21(315):1–11. 10.1186/s12905-021-01460-9 PMC8393774 34452616

[21] Commission on Social Determinants of Health [Internet]. Closing the gap in a generation: health equity through action on the social determinants of health. Geneva: World Health Organization; 2008. https://www.who.int/publications/i/item/9789241563703. 10.1016/S0140-6736(08)61690-6 18994664

[22] Social determinants of health and health inequalities [Internet]. Government of Canada; 2021. https://www.canada.ca/en/public-health/services/health-promotion/population-health/what-determines-health.html. Accessed 22 Oct 2021.

[23] Thurston W, Vissandjée B (2005). An ecological model for understanding culture as a determinant of women's health. Crit Public Health.

[24] Kennedy J. The importance of relationships in social work [internet]. Sussex: CareKnowledge; 2019. 15 p. https://www.careknowledge.com/media/44813/sw-and-relationships.pdf. Accessed 15 Oct 2021.

[25] Sandelowski M (2000). Whatever happened to qualitative description?. Res Nurs Health.

[26] Lambert VA, Lambert CE (2012). Qualitative descriptive research: an acceptable design. Pac Rim Int J Nurs Res.

[27] Bradshaw C, Atkinson S, Doody O (2017). Employing a qualitative description approach in health care research. Global Qual Nurs Res.

[28] Patton MQ (2015). Qualitative evaluation and research methods.

[29] Braun V, Clarke V (2006). Using thematic analysis in psychology. Qual Res Psychol.

[30] Fraser E. Impact of COVID-19 pandemic on violence against women and girls [internet]. London: The Violence Against Women and Girls Helpdesk. 16 p. 2020. https://www.sddirect.org.uk/media/1881/vawg-helpdesk-284-covid-19-and-vawg.pdf. Accessed 2 July 2021.

[31] Mittal S, Tingh S. Gender-based violence during COVID-19 pandemic: a mini-review. Front Glob Women’s Health. 2020;1(4). 10.3389/fgwh.2020.00004 PMC8594031 34816149

[32] Cravens J, Whiting J, Aamar R (2015). Why I stayed/left: an analysis of voices of intimate partner violence on social media. Contemp Fam Ther.

[33] Barriers to Leaving an Abusive Relationship [internet]. Center for Relationship Abuse Awareness; 2021. http://stoprelationshipabuse.org/educated/barriers-to-leaving-an-abusive-relationship/. Accessed 20 July 2021.

[34] Why Do Victims Stay? [internet]. National Coalition Against Domestic Violence; n.d. https://ncadv.org/why-do-victims-stay. Accessed 20 July 2021.

[35] Koepsell JK, Kernic MA, Holt VL (2006). Factors that influence battered women to leave their abusive relationships. Violence Vict.

[36] Grigsby N, Hartman BR (1997). The Barriers model: an integrated strategy for intervention with battered women. Psychotherapy.

[37] Davies S, Batha E. Europe braces for domestic abuse 'perfect storm' amid coronavirus lockdown [internet]. Thompson Reuters Foundation News, 2020. https://news.trust.org/item/20200326160316-7l0uf. Accessed 16 March 2022.

[38] Fuchs JD, Carter HC, Evans J (2021). Assessment of a hotel-based COVID-19 isolation and quarantine strategy for persons experiencing homelessness. JAMA Netw Open.

[39] Gray J. Could shelter hotels be a model for addressing homelessness? [internet]. The Globe and Mail, 2020. https://www.theglobeandmail.com/canada/article-could-shelter-hotels-be-a-model-for-addressing-homelessness/. Accessed 16 March 2022.

[40] Usher K, Bhullar N, Durkin J, Gyamfi N, Jackson D (2020). Family violence and COVID-19: Increased vulnerability and reduced options for support. Int J Ment Health Nurs.

[41] Mantler T, Veenendaal J, Wathen N (2021). Exploring the use of hotels as temporary housing by domestic violence shelters during COVID-19. Int J Homelessness.

[42] Tsioulcas A, Wamsley L. France announces plan to aid domestic abuse victims during Coronavirus crisis [internet]. NPR; 2020. https://www.nhpr.org/national/2020-03-31/france-announces-plan-to-aid-domestic-abuse-victims-during-coronavirus-crisis. Accessed 14 April 2020.

