# Supplementary material for: Service provider perspectives on how COVID-19 and pandemic restrictions have affected intimate partner and sexual violence survivors in Canada: a qualitative study
Source: BMC Womens Health. 2022 Apr 11;22:111. doi: 10.1186/s12905-022-01683-4 (PMC8996227; doi:10.1186/s12905-022-01683-4)
Supplement: Supplementary file 1 — Additional file 1. Interview Guide (Interview guide used with the 17 participants) [file 12905_2022_1683_MOESM1_ESM.docx]

Interview Guide

Note: Questions particularly relevant to the data subset reported on in this manuscript are highlighted in **bold underline**

**How has COVID-19 affected organizations such as yours, if at all? What have been the biggest challenges?**

How have employees personally been affected by COVID-19? How has it impacted their ability to carry out their duties?

**What are some of the changes organizations such as yours implemented, in order to carry out its mission in the current context of home confinement and social distancing?**

**Follow up: What impacts have had these changes on the women you serve?**

**Since the onset of COVID-19, have you noticed any changes in the needs of your clients?**

**Prompt: If so, what kind of changes? Can you give me some examples?**

**Since the onset of COVID-19, have you noticed a change in your clientele, or who is contacting your organization for support?**

**Prompt: If so, what kind of changes? Can you give me some examples?**

**Since the onset of COVID-19, have you noticed a change in the nature of violence experienced by women?**

**Prompt: If so, what kind of changes? (for instance, increased severity? A change in tactics?) Can you give me some examples?**

**Is there any sub-population of women who you think have been particularly impacted by the measures taken to contain the COVID-19 epidemic?**

Do you think other organizations working with women are facing similar challenges? What other challenges do you think they might be facing?

From your perspective, what do different types of organizations working in this field need to fulfill their mission right now and in the coming months?

Prompt: For example, financial, resources, human resources, more clarity on government measures, etc)

What challenges do you think your organization and similar agencies will face in the coming months?

What kind of data does your organization collect, if any, in terms of its clients and outreach?

Prompt: For instance, call volume, caller characteristics

From your perspective, how easy would it be to collect and compile such data?

Follow up: What logistical, legal, or ethical considerations are there in terms of sharing data with external people, such as researchers?

If we were to conduct a survey of IPV and sexual assault services across Canada, regarding the impacts of COVID-19, what data do you think would be important to collect? What data do you think would be feasible to collect?

What recommendations do you have for us, as researchers, in terms of how to approach and engage IPV and sexual assault services in completing a survey?

Is there anything else you would like to share?
